# Epigenetic control of tissue resident memory T cells

**DOI:** 10.3389/fimmu.2025.1605972

**Published:** 2025-08-15

**Authors:** Zhiyi Lan, Zeyu Chen, Nan Yang, Tong Liu, Siqi Li, Yuling Shi, Jun Gu

**Affiliations:** ^1^ Department of Dermatology, Shanghai Skin Disease Hospital, Tongji University School of Medicine, Shanghai, China; ^2^ Institute of Psoriasis, Tongji University School of Medicine, Shanghai, China; ^3^ Department of Dermatology, Shanghai Tenth People’s Hospital, Tongji University School of Medicine, Shanghai, China

**Keywords:** tissue resident memory T cells, epigenetics, chromatin accessibility, DNA methylation, histone modification, non-coding RNAs

## Abstract

Tissue-resident memory T cells (TRM) represent a heterogeneous population of T cells that exhibit both effector and memory functionalities. They express specific gene signatures that enable them to occupy tissues without recirculating, thus providing a first response against reencountered pathogens or antigens. TRM have been implicated in the pathogenesis of various diseases, including autoimmune disorders, infections, and cancers. This has prompted interest in targeting TRM as a potential therapeutic strategy. Epigenetic modifications, which frequently occur in immune cells across various disease states, play a significant role not only in tissue homeostasis but also in disease progression. Emerging evidence suggests that the epigenetic landscape of TRM is altered in pathogenic conditions, impacting their differentiation, maintenance, and function. Nevertheless, the precise mechanisms remain poorly understood. This review seeks to provide a comprehensive overview of the epigenetic regulation of TRM, focusing on key areas such as chromatin accessibility, DNA methylation, histone modifications, and non-coding RNAs. Importantly, a deeper understanding of these epigenetic mechanisms will pave the way for novel therapeutic strategies, such as modulating TRM activity in autoimmune diseases, enhancing tissue-specific immunity through vaccines, or improving immunotherapeutic efficacy in cancer.

## 1 Introduction

lymphocytes are the core component of the adaptive immune system, essential for various functions including infection resistance, anti-tumor immunity, and immune regulation (1). Recent years, an increasing number of studies have focused on a distinct lineage of memory T cells which was defined as tissue-resident memory T cells (TRM) (2). Distinct from central memory T cells (TCM) and effector memory T cells (TEM) which engage in the recirculation through the blood and lymphoid organs, TRM are primarily localized to specific organs such as skin, lung, liver, and small intestine (3). This residency property mainly ascribes to low expression of C-C chemokine receptor type 7, sphingosine-1-phosphate receptor 1 (S1PR1) (4) and CD62L, which are critical for the egress of TCM and TEM from peripheral tissues. Notably, TRM are not a single entity. Despite considerable overlap, CD4^+^and CD8^+^TRM exhibit lineage-specific features and regulatory differences that are influenced by both the tissue niche and the disease setting (5). There is substantial phenotypic heterogeneity, but TRM commonly express specific cell surface markers such as C-type lectin CD69 and integrin α E (CD103), which facilitate their maintenance in tissues and play a role in modulating their functional responses (2). Due to this specificity, TRM play a first-line role in the body’s resistance to foreign pathogen invasion and anti-tumor immunity (6). However, recent studies have highlighted that dysregulation or excessive activation of TRM can contribute to autoimmune diseases (7–9). Thus, TRM are considered as a potential target of several chronic recurrent inflammatory diseases, most notably inflammatory bowel disease, psoriasis and rheumatoid arthritis. Due to the importance of TRM in disease onset and relapse, it is necessary to explore the detailed mechanism of TRM regulation in order to find a viable way to cure diseases.

Regulation mechanisms of TRM encompass various aspects of cellular life cycle including formation, maintenance, and function in the previous studies (2). Thus far, substances like transcription factors, cytokines, tissue-specific cell adhesion molecules and anatomic compartments are well-established influencing factors in the regulation processes of TRM (10). Recent study have demonstrated that TRM display a unique epigenetic landscape amidst memory T cell subsets (11). However, the epigenetic characteristics behind all these influencing factors have not received as much attention. Understanding these epigenetic characteristics could provide deeper insights into how TRM are regulated and may reveal novel avenues for therapeutic intervention. Emerging evidence indicates that the tissue microenvironment plays a pivotal role in shaping the epigenetic landscape of TRM. Local cues such as cytokines, metabolites, and stromal cell-derived signals can influence chromatin accessibility, DNA methylation, and histone modifications in TRM and their precursors, thereby guiding their differentiation and maintenance within specific tissues (12). These microenvironmental signals integrate with intrinsic transcriptional programs to generate distinct, tissue-adapted epigenetic profiles, underpinning the functional heterogeneity of TRM across different organs.

The central dogma of molecular biology posits that DNA undergoes self-replication, transcription into RNA, and subsequent translation into proteins (13). Each of these processes is regulated by various factors, which can modulate the expression levels of specific proteins. This regulatory mechanism which enables cells with identical DNA sequences to undergo differential development and differentiation is referred to as epigenetic modification (14). Divergent from genetic changes, epigenetic modifications regulate gene expression and cellular phenotypes dynamically without altering the DNA sequence itself. These modifications can influence gene activity and can be inherited across generations, thereby impacting cellular functions and traits (15). The most recognized epigenetic regulations are DNA methylation, histone modifications, non-coding RNA-mediated regulation, among others (16). Together, these alterations dynamically regulate chromatin structure and accessibility, ultimately fine-tuning gene expression. This process shape the unique gene expression profile of TRM, enabling them to adapt to the specific tissue microenvironment and exert corresponding functions (17). Moreover, emerging evidences indicate that the molecular mechanisms associated with the epigenetics of TRM could present novel targets for cancer immunotherapy and the treatment of autoimmune diseases. This review aims to summarize recent advances in how distinct epigenetic mechanisms—including chromatin accessibility, DNA methylation, histone modifications, and non-coding RNAs—individually contribute to the differentiation, maintenance, and functional regulation of TRM. Each section is structured to follow this biological sequence, discussing relevant epigenetic mechanisms accordingly.

## 2 Chromatin accessibility

Chromatin accessibility refers to the degree to which chromatin, the complex of DNA and proteins in the nucleus, is open and accessible for transcriptional machinery, regulatory proteins, and other factors involved in gene expression. This accessibility is often regulated by epigenetic mechanisms, including DNA methylation, histone modifications, and non-coding RNAs. Recent studies indicate that chromatin accessibility is a crucial determinant of TRM identity and functionality, influencing their response to local microenvironments. First, the chromatin accessibility landscape of TRM diverge from those of circulating T cells (18, 19). Current studies predominantly focus on CD8^+^T cells, with limited attention given to their CD4^+^counterparts. The assay for transposase-accessible chromatin with sequencing (ATAC-seq) analysis on day 7 post-infection revealed that CD8^+^T cells in non-lymphoid tissues, such as intraepithelial lymphocytes (IEL) TRM precursors, exhibit distinct chromatin profiles compared to splenic effector cells. Regions near TRM-associated genes, such as *Cd69*and *Nr4a1*, were accessible, while regions near recirculation-associated genes, such as *Klf2*and *S1pr1*, were less accessible (18). A recent study establishing a multiomic single cell atlas of antigen-specific CD8^+^T cell states across infection and cancer reveals the unique transcriptional and epigenetic features of TRM. In this study, tumor-infiltrating lymphocytes and TRM shared unique changes in the expression of 293 genes and accessibility patterns of 796 regions compared with other antigen-specific T cells, suggesting significant alterations in chromatin accessibility in tissue-resident T cells. Further analysis demonstrated that the transcription factor KLF2 likely repressed accessibility and expression of residency-related genes, while BATF likely enhanced accessibility at loci associated with TRM characteristics (19). Second, TRM exhibit distinct chromatin accessibility profiles depending on their tissue environment. In one study, 7,150 differentially accessible regions (DARs) were identified when comparing splenic circulating P14 cells to TRM from the IEL, kidney, salivary glands (SG), fat, and liver following lymphocytic choriomeningitis virus (LCMV) infection. Clusters of accessible genomic regions were classified into tissue-specific, tissue-shared, broadly circulating, and broadly resident profiles. For example, the TRM marker *Itgae*showed increased accessibility at its transcription start site (TSS) in IEL and SG TRM, while the circulation-associated gene *Sell*was more accessible in splenic T cells. A uniquely accessible TSS region of *Ccr9*was identified specifically in IEL TRM (17). Furthermore, chromatin accessibility also distinguishes TRM subsets within the same tissue. A study revealed that intestinal CD8^+^TRM clustered distinctly based on differential chromatin accessibility. Four different parts of intestinal tissue were analyzed using cellular indexing of transcriptomes and epitopes. Uniform manifold approximation and projection (UMAP) analyses revealed that TRM from each intestinal tissue compartment formed distinct clusters; however, small intestine intraepithelial and lamina propria TRM clustered more closely with each other, as did colon intraepithelial and lamina propria TRM. Additional heterogeneity within and among TRM populations from each intestinal compartment was also observed (20). In addition, Chromatin accessibility profiles also distinguish different skin CD8^+^TRM subsets (21). To investigate epigenetic differences among CD8^+^TRM subsets in human skin, ATAC-seq was performed on four major populations defined by CD103 and CD49a expression, identifying over 100,000 chromatin-accessible regions, most of which were located in enhancer regions. Principal component analysis showed clear separation between dermal and epidermal TRM, with dermal cells displaying the most distinct chromatin features. Epidermal CD103^+^CD49a^+^TRM had unique accessible regions near genes linked to cytotoxicity (e.g., *GZMB*, *PRF1*, and *IFNG*) and TCR/NK signaling (21).

While chromatin accessibility profiling reveals the static epigenetic landscapes that define TRM across tissues and subsets, recent studies have also begun to uncover the upstream mechanisms that dynamically shape these accessible regions. Notably, extrinsic signals from the tissue microenvironment, such as those derived from fibroblastic reticular cells (FRCs) and IL-6, have been shown to actively remodel chromatin accessibility during the early stages of CD8^+^T cell activation, thereby influencing TRM differentiation. FRCs along with IL-6 can enhance cytokine production of IL-2 and Tumor Necrosis Factor-α (TNF-α) and remodel chromatin accessibility lead to the upregulation of 778 genes through driving greater accessibility at 402 chromatin-accessible regions in newly activated CD8^+^T cells. Signals from FRCs have been shown to enhance the accessibility of transcription factor binding motifs such as MYC, HIF-1α, and HIF-1β, which are associated with the activation of metabolic pathways following T cell activation. Additionally, FRC-derived factors promote the enrichment of motifs for key transcription factors involved in CD8^+^T cell survival and memory differentiation, including BATF, ETS1, and BACH2. That would induce profound metabolic reprogramming in CD8^+^T cells, including enhanced glycolysis, increased oxidative phosphorylation, and upregulation of lipid synthesis and storage pathways. These metabolic features closely resemble the energy profiles observed in memory CD8^+^T cells and TRM, leading to epigenetic changes that facilitate their differentiation into TRM Chromatin-accessible regions induced by both FRCs and IL-6 conditioning were significantly enriched for memory precursor-specific epigenetic modifications, suggesting a priming effect on the transcriptional landscape of CD8^+^T cells. Consistently, FRC-educated CD8^+^T cells exhibited markedly improved persistence at both early (day 8) and later (day 37) stages following influenza infection, and preferentially differentiated into TRM by day 143. This result indicates that FRC-derived signals could drive metabolism-associated epigenetic changes that support CD8^+^T cell survival and memory differentiation in tissues (22).

## 3 DNA methylation

DNA methylation, which plays a pivotal role in the epigenetic mechanism of the mammalian genome, involves the modifications of cytosine by add a methyl group to the fifth carbon to form 5-methylcytosine (5mC) (23). It participates in various biological processes, including genomic imprinting, X-chromosome inactivation, and repression of transposable elements and germline-specific genes, by which it influences the courses of gene expression, cell differentiation, and development (24). Despite its capability to activate transcription in certain cases, DNA methylation is more commonly regarded as repressor of gene expression through different mechanisms (23). One of the most established mechanisms involves the methylation of CpG islands within gene promoters. Although not fully understood, this process can interfere with the binding of transcription factors, ultimately impacting cellular function and development through gene silencing (25). Some research has indicated that this mechanism exerts influence in the modulation of TRM.

Previous studies have shown that both CD4^+^and CD8^+^T cells exhibit distinct DNA methylation patterns in key genes that determine their cell fate (26). During acute LCMV infection, memory precursor cells undergo dynamic methylation changes: effector-associated genes such as *Ifng, Prf1*, and *Gzmb*become demethylated and expressed, while naïve-associated genes are initially methylated. Interestingly, a subset of these repressed naïve genes is later demethylated and re-expressed in memory T cells, reflecting epigenetic flexibility (27). In addition, DNA methylation of the *Gzmb*gene serves as a useful marker to distinguish memory CD4^+^Th1 cells from T follicular helper (Tfh) cells (27). Deletion of *Dnmt3a*, a *de novo*DNA methyltransferase, accelerates memory formation by preventing the stable silencing of memory-favoring genes (28). Conversely, deficiency of TET2, a DNA demethylase, also enhances memory generation, but through increased methylation of transcription factors (e.g., *Tbx21*, *Prdm1*, *Runx3*) that drive effector differentiation, indirectly skewing the fate toward memory (29). The transcription factor FOXP1, which regulates memory T cell differentiation, is subject to DNA methylation control, with a progressive loss of methylation observed during the transition to memory cells. Furthermore, the expression of *Foxp3*in memory regulatory T cells (Tregs) is regulated by DNA methylation at the Treg-specific demethylated region (30). As a subtype of memory T cells, TRM also exhibit different DNA methylation landscape. In the differentiation of CD4^+^memory T cells, a bone-marrow resident T cell population was observed to diverge early from the main pathway (naïve T cells-TCM-TEM-terminally differentiated TEM), exhibiting a distinct epigenomic profile, as revealed by comprehensive transcriptome analyses, primarily including genome-wide DNA methylation profiles. These findings suggest that acquisition of a resident phenotype is linked to extensive DNA methylation reprogramming (31).

DNA methylation patterns are also altered in key functional genes of memory T cells. Lung CD8^+^TRM play a paramount role in viral reinfection (32). A study has demonstrated that the expression of the transmembrane protein Interferon-induced transmembrane protein 3(IFITM3), which facilitates survival and resistance to viral infection during subsequent exposures in CD8^+^TRM, is regulated by DNA methylation. As one of main methods to identify DNA methylation, Bisulfite sequencing was applied to detect the CpG island in the *Ifitm3*promoter in genomic DNA extracted from CD8^+^and CD4^+^TRM from mice recovered after influenza virus infection (33). The results showed that constitutive expression of IFITM3 in CD8^+^rather than CD4^+^TRM correlates with hypomethylation of the IFITM3 promoter 20 days after infection with influenza virus (33). However, IFITM3 expression was found to be upregulated in lung TRM as early as 10 days post-influenza infection and remained elevated for at least 60 days, indicating a long-term hypomethylated state of the *IFITM3*promoter. Upregulation of IFITM3 was not found in effector T cells, suggesting that demethylation of IFITM3 plausibly occurs during the effector-to-memory transition. Notably, IFITM3 upregulation was induced either by recognition of the cognate antigen or by exposure to interferon-α (IFN-α). Nevertheless, these stimuli are not responsible for maintaining sustained IFITM3 expression, as IFN-α was undetectable in the lungs after the resolution of acute infection at 10 days post-influenza virus clearance. The expression of *IFITM3*is essential for the functional integrity and long-term survival of lung TRM, which not only mediate protection against the primary pathogen that induced their formation but also confer resistance to other cytopathic viruses encountered in peripheral tissues. Meanwhile, *IFITM3*expression may not be a phenomenon restricted to the lung. Elevated *IFITM3*expression is also a characteristic feature of brain TRM generated during vesicular stomatitis virus (VSV) infection, although it remains unclear whether this is a consequence of hypomethylation at the *IFITM3*promoter (34). Similarly, this kind of demethylation has also been observed in tumors. TRM demonstrate ascendant cytotoxic capability in human urinary bladder cancer (UBC) when compared to other types of T cells, such as TCM (35). This phenomenon occurs due to the less methylated locus in the *Perforin1*(*PRF1*) reporter CpG site (–1053), identified in the enhancer region of *PRF1*in CD8^+^TRM (35). The hypomethylation at this locus leads to elevated expression of perforin, a protein that perforates the target cell membrane, facilitating the entry of granzyme and promoting apoptosis of tumor cells (35). However, no significant difference in DNA methylation at the PRF1 locus was observed between patients with non-muscle-invasive (stage I) and those with muscle-invasive (stage ≥II) tumors, suggesting that *PRF1*remains transcriptionally active across both early and advanced tumor stages. In another research, during Simian Immunodeficiency Virus (SIV) infection, promoter-wide and CpG-site specific methylation increased at *Ifng*and *Il2*promoters but decreased at the *Tnf*promoter in intestinal tissue-derived CD8^+^T cells, although CD8^+^TRM were not tested directly (36). Taken together, DNA methylation plays a crucial role in enhancing cytotoxicity against infected or cancer cells by influencing the expression of key genes that regulate the function of TRM. However, further research is needed to understand how alterations in DNA methylation at key gene loci impact TRM commitment and function. Besides, the role of other DNA methylation modifiers, such as DNMT1 and TET1/3, in TRM remains undefined. DNMT1 maintains DNA methylation essential for T cell survival, lineage fidelity, and controlled cytokine expression. Its loss disrupts TCRαβ development, induces aberrant CD8^+^TCRγδ^+^cells, and enhances cytokine gene activation via demethylation (37). TET1 and TET3 regulate epigenetic programs critical for CD8^+^T cell maintenance by mediating DNA demethylation at survival- and metabolism-related genes. Their deletion results in increased apoptosis, impaired IL-7 signaling, and reduced peripheral CD8^+^T cells (38). Therefore, elucidating their regulatory function in TRM is of great importance.

## 4 Histone modifications

Chromatin refers to the intricate combination of DNA and histone proteins present within the nucleus of eukaryotic cells, serving as the structural foundation for the organization of the entire genome. The fundamental building block of chromatin is the nucleosome, comprising 147 base pairs of DNA intricately wound around a histone octamer composed of two instances each of histones H2A, H2B, H3, and H4 (39). Although histone modifications can occur at the core residues of histone proteins, the majority of modifications, such as acetylation, methylation, phosphorylation, ubiquitination, sumoylation, adenosine diphosphate ribosylation, and deamination, primarily take place on the more flexible and exposed histone tails. The histone tails extend from the nucleosome core and are more accessible to the enzymes responsible for adding or removing these chemical groups. After these alterations, chromatin compaction and accessibility can be influenced which ultimately changes the state of transcription (40). Although there exist several kind of modifications, histone acetylation and histone methylation are the most extensively studied (41).

TRM differentiation can be regulated by histone modifications in the context of inflammatory diseases and foodborne pathogen infection. Inflammatory Bowel Diseases (IBD) are characterized by chronic inflammation of the gastrointestinal tract. TRM play a central role in the pathogenesis of IBD (42). Recent studies have demonstrated that the insulin receptor of intestinal mucosal T-cells can promote intestinal CD4^+^TRM differentiation via Enhancer of zeste homolog 2 (EZH2) (43). EZH2 is a key catalytic subunit of the Polycomb repressive complex 2, which functions by catalyzing the trimethylation of Lys-27 on histone 3, leading to the alteration of gene expression (44). Barrier disruption and pathogen invasion trigger the expansion of effector T cells in the gut. Insulin signaling enhances EZH2 expression, which facilitates these effector T cells differentiation into TRM through H3K27 trimethylation. This epigenetic regulation is thought to involve genomic regions controlling core transcription factors such as Hobit and Blimp1, although the precise targets remain to be fully elucidated (43). Another study on intestinal CD8^+^TRM reveals that histone modifications regulated by key transcription factors are crucial for their tissue residency and functional capacity in a *Listeria monocytogenes*infection model. The transcription factor B cell leukemia/lymphoma 11B (Bcl11b), which acts both as a transcription repressor and as a transcription activator, plays a crucial role not only in thymocyte development but also in the differentiation and function of mature T cells (45). It has been considered as a frontrunner in CD8^+^TRM programs. Bcl11b effectively maintains the expression of multipotent/multifunctional (MP/MF)-related genes in TRM while suppressing the expression of effector program genes, suggesting its role in preserving the developmental and functional potential of TRM (46). By conducting Bcl11b Chromatin Immunoprecipitation Sequencing (ChIP-seq), H3K27ac ChIP-seq and H3K4me3 Cleavage Under Targets and Release Using Nuclease on TRM-like cells, Bcl11b was found to regulate the epigenetic landscape and directly controlling expression of essential genes of the MP/MF program. Bcl11b enhances H3K27ac, H3K4me3, and chromatin accessibility at the promoter regions of various MP/MF-related genes, such as *Transcription Factor 7(Tcf7), Inhibitor of DNA binding 3(Id3).*Recent study found that small intestine CD8^+^TRM expressing *Tcf7*and high levels of *Id3*represent a subset with enhanced memory potential (47, 48). This suggests that Bcl11b is necessary for maintaining H3K27ac, H3K4me3, and chromatin accessibility at the promoter regions of these genes, thereby sustaining their expression and preserving the memory potential of CD8^+^TRM. Additionally, Bcl11b can suppress the expression of effector program genes, including *Prdm1*and *Ahr*, restricting CD8^+^TRM differentiation (46). Further research focusing on histone modifications regulated by key transcription factors is needed to advance therapies targeting TRM through these mechanisms.

In muscle-invasive bladder cancer, RNA-seq data show that tumors with high infiltration of CD103^+^CD8^+^TRM exhibit significantly more frequent alterations in the gene *lysine methyltransferase 2A*(KMT2A) compared to tumors with low TRM infiltration (49). Conversely, mutations in the gene *lysine demethylase 6A*(KDM6A) are more prevalent in the low TRM infiltration subgroup (49). The proteins products of these two genes respectively function as the methyltransferase and demethylases towards histone tails, particularly the histone H3 tail. Both enzymes are crucial for chromatin dynamics, which influence the accessibility of genes for transcription (50, 51). KMT2A has not been functionally defined in T cell biology. KDM6A, a sex chromosome, is enriched in Th17 cells from female patients with ankylosing spondylitis and is associated with a pro-inflammatory transcriptional profile (52). Although these findings suggest a correlation between histone modification gene mutations and TRM abundance, direct mechanistic evidence linking KMT2A or KDM6A to TRM differentiation, migration, or retention in tumors is currently lacking. To further establish causality, future studies could employ Clustered Regularly Interspaced Short Palindromic Repeats (CRISPR)-mediated knockout or pharmacological inhibition of KMT2A/KDM6A in tumor-infiltrating CD8^+^T cells, followed by assessment of TRM phenotype and localization *in vivo*. Additionally, tumor organoid models co-cultured with TRM precursors or adoptive TRM transfer systems into genetically engineered mouse models could provide tractable platforms to explore how these epigenetic regulators shape TRM residency and function. Given that KDM6A is X-linked and escapes X inactivation, it may exhibit sex-specific regulatory effects on T cells, contributing to differential TRM responses (53). Future studies using sex-matched T cells or four-core genotype mouse models will be valuable to dissect its dosage- and context-dependent immunological roles. This finding raise the possibility that changes of histone modification may influence the infiltration of TRM in the tumor tissue thus make an impact to the prognosis of muscle-invasive bladder cancer (49).

Recently, emerging evidence has highlighted the regulatory role of histone modifications in modulating the functional activities of TRM, particularly in the contexts of tumor immunity and inflammatory diseases. Basic helix-loop-helix transcription factor 40 (Bhlhe40) is a stress-responsive transcription factor that is important for numerous cell physiological responses. Bhlhe40 functions to promote mitochondrial gene transcription in TRM. Mitochondria play key roles in the biosynthesis and epigenetic regulation of gene expression. The deficiency of *Bhlhe40*leads to reduction in metabolites in the tricarbox ylic acid (TCA) cycle, butanoate metabolism, amino acid (aa) metabolism. These metabolites involved in acetyl coenzyme A synthesis, which is a critical substrate of histone acetylated modification in CD8^+^T cells including TRM (54). Conversely, enforced expression of *Bhlhe40*modestly enhanced the expression of mitochondrial respiratory chain genes and promoted histone H3 acetylation, suggesting that Bhlhe40 may regulate histone acetylation at functional gene loci in CD8^+^T cells. In accordance, Bhlhe40 deficiency attenuates the function of the CD8^+^TRM by decreasing the acetylation in H3K9 and H3K27 in the *Ifng*locus in an acute influenza virus infection model (54). Subsequently, addition of the downstream products of Bhlhe40, especially tubastatin A and acetate, could restore the interferon (IFN) -γ production by CD8^+^T cells that lack Bhlhe40, as well as increase CD69 and CD103 expression through increasing histone H3 acetylation, thus promoting the TRM residency and function of resistance to tumors (54). This study suggests that gene deficiency can also lead to epigenetic changes, although in most cases, it is the epigenetic alterations that regulate the expression of critical genes, thereby influencing the residency and function of TRM. In addition, epigenetic modification sometimes correlates with metabolism in TRM. Appropriate combinations of epigenetic modulators and specific metabolites may represent promising strategies to optimally reinvigorate the antiviral or antitumor functions of tissue- or tumor-resident CD8^+^T cells.

Similar to DNA methylation modifiers, several key histone-modifying enzymes remain poorly studied in the context of TRM. HDAC1 plays distinct, context-dependent roles in T cell development and lineage stability. It is essential for thymocyte maturation, peripheral T cell homeostasis, and antiviral CD8^+^T cell responses. Deletion of HDAC1 impairs the transition from immature CD8^+^CD4^-^thymocytes to the double-positive stage and reduces the expansion of virus-specific CD8^+^T cells, despite enhanced IFN-γ production by effector cells (55). Recent evidence also implicates HDAC1 in regulating exhausted CD8^+^T cell fate during chronic viral infection (56). In parallel, HDAC1 together with HDAC2 maintains CD4^+^T cell lineage integrity by repressing CD8-lineage genes such as *Cd8a*and *Cd8b1*. Loss of HDAC1/2 in CD4^+^T cells leads to aberrant activation of a CD8^+^effector-like program via Runx–CBFβ complexes, particularly in Th0 and Th1 subsets (57). Moreover, HDAC1 could promote CD4^+^T cell hyperactivation in systemic lupus erythematosus by repressing *microRNA-124*(miR-124) expression through promoter binding. Reduced miR-124 results in the upregulation of *interferon regulatory factor 1*, thereby enhancing T cell immunoactivity (58). These findings highlight HDAC1 as a critical epigenetic regulator with diverse functions in T cell biology, including development, lineage fidelity, and immune dysregulation in autoimmunity. HDAC3 acts as a transcriptional repressor of chemokines such as CXCL10 in KRAS-mutant lung cancer cells, limiting T cell recruitment to the tumor microenvironment. Its inhibition enhances chemokine expression via an NF-κB/p65-dependent pathway and promotes T cell infiltration into lung tumors *in vivo (* 59). HDAC4 appears to play a limited role in T cell immunity under steady-state conditions. Although it is expressed in multiple T cell lineages, T cell-specific HDAC4 deficiency does not alter the frequencies or cytokine production of conventional T cells, *i*NKT cells, or regulatory T cells (60). Given their established roles in regulating T cell activation, lineage differentiation, and tissue-specific transcriptional programs, HDACs—particularly HDAC1—may also participate in the formation, maintenance, or functional adaptation of TRM. Whether these enzymes modulate TRM epigenetic identity or residency-related gene expression remains to be determined, warranting further investigation.

## 5 Non-coding RNAs

Non-coding RNAs (ncRNAs) are RNA molecules that lack the genetic information necessary for protein production (61). Of the three billion base pairs in the human genome, only 2% are responsible for encoding proteins, while the remainder consists of ncRNAs (62). NcRNAs exhibit significant heterogeneity in their length, conformation, and cellular functions. Recent taxonomy distinguishes between long non-coding RNA (lncRNA) and small non-coding RNA. Within lncRNA, distinctions can be made between linear RNAs and circular RNAs. Moreover, each group can be subdivided into tens of thousands of specific ncRNAs, all of which play vital roles in various physiological and pathological processes (63).

Several studies have explored the relationship between different ncRNAs and various types of T cells, including CD4^+^T cells, CD8^+^T cells, regulatory T cells, and others (64). Among ncRNAs, miRNAs, lncRNAs and circular RNAs (circRNAs) are the most well-studied subtypes which were found to impact the differentiation (65, 66) and function (67, 68) of T cells. However, the regulation of TRM by ncRNAs was not well investigated.

MiRNAs consist of short RNA molecules, typically about 22 nucleotides in size and are produced by two Ribonuclease III proteins, Drosha and Dicer (69). They play a role in posttranscriptional silencing of target genes, with each miRNA capable of targeting a multitude of messenger RNAs. This process affects the expression of numerous genes, particularly those involved in functional interacting pathways (70). Various miRNAs are involved in the regulation of T cell function. MiR-155 plays a crucial role in T cell-mediated immunity by promoting Th1 and cytotoxic T cell responses and is essential for T cell activation, cytokine production, and immune memory (71, 72). MiR-146a acts as a negative regulator of T cell-driven inflammation by suppressing NF-κB signaling through targeting Traf6 and Irak1, and it is vital for Treg function and the control of Th1-mediated immune responses (73, 74). The miR-17~92a cluster enhances T cell activation and Th1 differentiation by promoting IFN-γ production (75). MiR-181 is critical for thymocyte development and TCR sensitivity by modulating both positive and negative selection processes (76). Although miRNAs have been extensively studied in T cells, only miR-155 and miR-181a have been directly investigated in TRM. MiR-155 is a class of miRNAs produced by lymphoid cells, myeloid cells, and bone marrow blasts, exerting its influence on immune cell proliferation and differentiation, as well as on the regulation of innate and adaptive immune responses, inflammation, and carcinogenesis (77, 78). MiR-155 has been found to closely correlate with the fate of CD4^+^T and CD8^+^T cells, partly through its posttranscriptional regulation of multiple target genes (79, 80). A recent study investigated the role of miR-155 in the development of TRM by infecting mouse brains with a lethal dose of *Listeria monocytogenes*. Following the infection, mice were treated with either antibiotics combined with a miR-155 inhibitor or antibiotics combined with a scramble control. It was found that the application of miR-155 inhibitor led to reduced accumulation of brain CD8^+^TRM compared to the scramble group (81). Meanwhile, numbers of brain CD8^+^TRM in infected *miR-155* ^-/-^mice were also significantly reduced compared to infected wild-type mice (81). These results suggest that miR-155 is critical for the maintenance of TRM. MiR-181a, initially discovered in murine thymocytes and T cells, serves as a pivotal modulator of the T cell receptor activation threshold (82). It exhibits high expression levels in double-positive thymocytes, yet undergoes a decremental trend in single-positive thymocytes and peripheral T cells (76). This distinctive expression profile is posited to expedite positive selection via autoantigen recognition while concurrently mitigating the risk of autoimmunity (83). A recent study found that mature CD8^+^T cells with miR-181 deficiency failed to acquire a tissue-resident phenotype in the liver following infection with lymphocytic choriomeningitis virus (84). Importantly, this effect was observed not only during acute infection but also persisted into later memory time points (84). Taken together, current research predominantly focuses on miRNAs, while other ncRNAs with potential regulatory roles in TRM remain underexplored. MiR-155 and miR-181a play distinct tissue-residency roles in TRM, which differ from their functions in conventional T cells. This suggests that other types of miRNAs may also possess unique properties in TRM, making this a promising area for future research.

LncRNAs are a diverse and underexplored class of regulatory molecules that have emerged as key modulators of gene expression in various branches of the immune system (85, 86). A lot of studies have been conducted to investigate the regulatory role of lncRNAs in T lymphocytes. Distinct lncRNAs modulate different intracellular signal transduction pathways across specific T cell lineages, participating in key biological processes such as development, differentiation, activation, and effector functions (87). A microarray analysis of mouse naïve, memory, and effector CD8^+^T cells identified 1,106 expressed lncRNAs, with 10% showing differential expression, including 21 associated with naïve-to-memory cell differentiation. The lncRNA Morrbid, induced by lymphocytic choriomeningitis virus infection or T cell receptor stimulation in mouse CD8^+^T cells, is required for the upregulation of Bcl2l11—an apoptosis-inducing factor essential for CD8^+^T cell contraction—in both CD4^+^TCM and TEM following *in vitro*stimulation. These studies suggest that lncRNAs may also promote the acquisition of TRM memory characteristics, although their roles remain to be experimentally validated (88, 89). CircRNAs are stable single-stranded RNA molecules that form a covalently closed continuous loop (90). A recent study demonstrated that circular circRNAs are differentially expressed during thymocyte differentiation, which can be categorized into three main stages: early immature (ST1; CD34^+^CD2^-^), intermediate (ST2; CD1A^+^), and mature (ST3; CD1A^-^) thymocytes (91). Among these, hsa_circIKZF1_0001, which was previously identified as a T cell-specific circRNA in mature blood cell populations, exhibited a progressive increase in expression throughout thymocyte development (92). In contrast, hsa_circHIPK3_0001, which has been reported to modulate cell growth and proliferation in various human cell types, showed a decreasing expression pattern during differentiation (93). As we know, circRNAs regulate mRNA expression by acting as sponges for specific miRNAs. Notably, the downregulation of *RAG2*, a gene essential for V(D)J recombination during early T cell development, from ST1 to ST3 stages may be mediated by the reduced expression of hsa_circ_0031584 (*ARHGAP5*) and hsa_circ_0019079 (*KIF20B*), potentially through the release of hsa-miR-609. These results indicated that circRNAs get involved in T-cell differentiation in the thymus through circRNA–miRNA–mRNA networks (91). Future experimental validation is required to establish the involvement of circRNAs in regulating genes critical for T cell differentiation in the thymus, with some circRNAs potentially playing important roles in TRM cell fate determination. However, it is important to note that while these findings suggest potential regulatory roles of lncRNAs and circRNAs in T cell biology, there is a lack of direct experimental evidence demonstrating their functions specifically in tissue-resident memory T cells. To address this gap, future studies could apply targeted loss- or gain-of-function approaches, such as *in vivo*CRISPR interference or CRISPR activation, to modulate candidate lncRNAs or circRNAs in tissue-resident CD8^+^T cells. Such experiments would enable functional validation of these non-coding RNAs in TRM differentiation, maintenance, and effector function within their physiological tissue niches.

## 6 Discussion and perspectives

**Figure 1:**
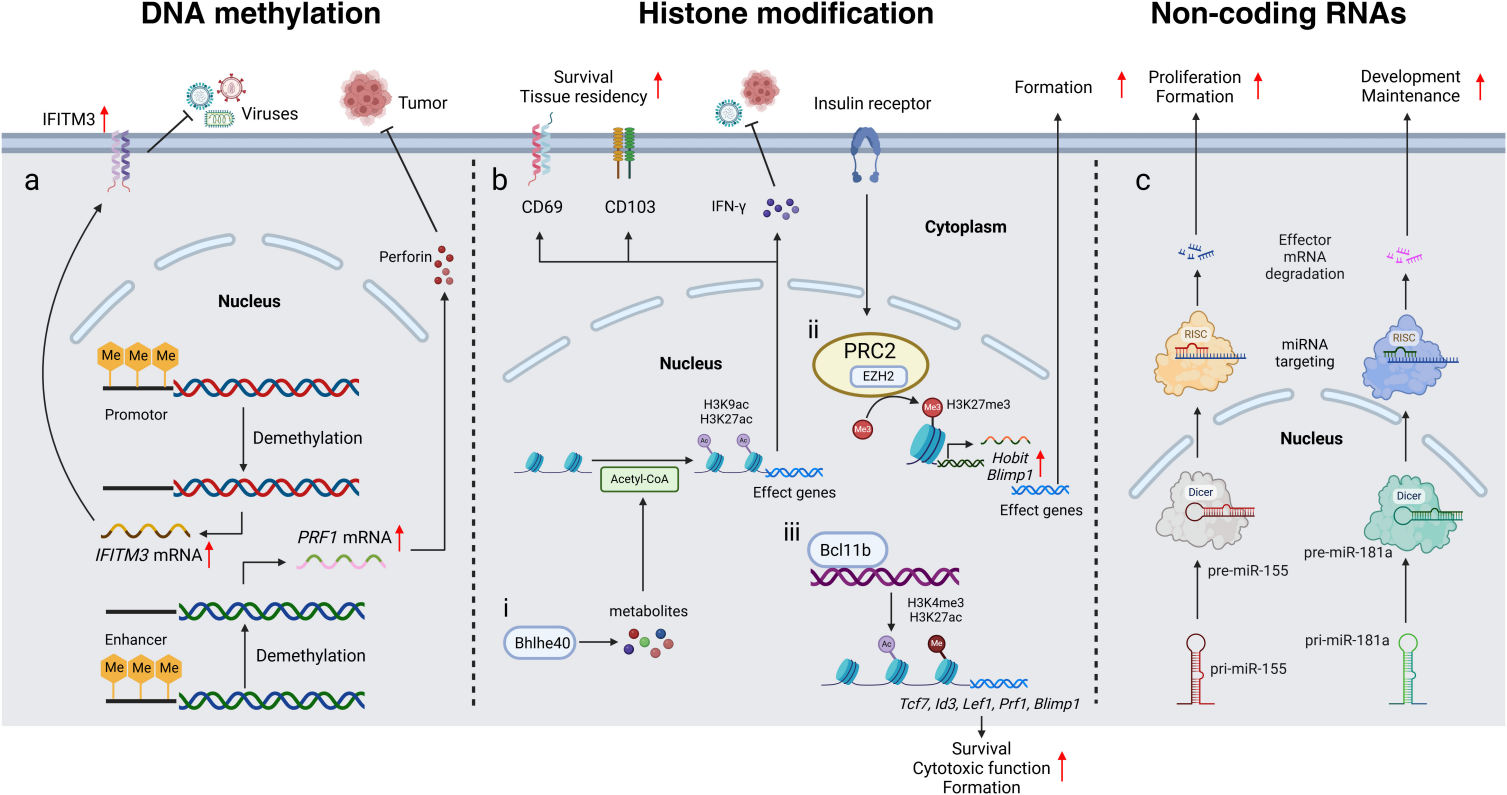
Epigenetic control of tissue resident memory T cells. **(a)** Demethylation of the *IFITM3*promoter and the *PRF1*enhancer increases gene expression, thereby facilitating antiviral defense and tumor resistance, respectively. **(b)** i. Bhlhe40 enhances the production of metabolites involved in acetyl-CoA synthesis, thereby facilitating H3K9 and H3K27 acetylation at the *Ifng*locus and other gene loci. This leads to increased secretion of IFN-γ, as well as upregulated expression of CD69 and CD103, ultimately promoting tumor resistance, antiviral defense, TRM survival, and tissue residency. ii. PRC2, with its key catalytic subunit EZH2, promotes the tri-methylation of H3K27 (H3K27me3), thereby facilitating TRM formation by regulating the expression of core transcription factors such as Hobit and Blimp1. iii. Bcl11b facilitates H3K27ac and H3K4me3 at the promoters of MP/MF program genes, as well as *Prf1*and *Blimp1*, promoting TRM survival, cytotoxic function and differentiation. **(c)** Pri-miR-155 and pri-miR-181a are transcribed in the nucleus and subsequently processed by Dicer into pre-miRNAs. These pre-miRNAs are then exported to the cytoplasm, where they are incorporated into RISC complexes. Within the RISC complex, miRNA targeting occurs, leading to effector mRNA degradation or translational repression. Through this process, miR-155 drives TRM proliferation and formation, while miR-181a supports their formation and maintenance. *IFITM3*Interferon-Induced Transmembrane Protein 3*, PRF1*Perforin 1*, Me*Methyl group*, CD69*Cluster of Differentiation 69*, CD103*Cluster of Differentiation 103*, IFN-γ*Interferon Gamma*, PRC2*Polycomb Repressive Complex 2*, EZH2*Enhancer of Zeste Homolog 2*, Acetyl-CoA*Acetyl Coenzyme A*, Bhlhe40*Basic Helix-loop-helix Transcription Factor 40*, H3K27me3*Trimethylation of Lysine 27 on Histone H3*, Bcl11b*B Cell CLL/Lymphoma 11B, *MP/MF*multipotent/multifunctional, *Tcf7*Transcription Factor 7*, Lef1*Lymphoid Enhancer Factor 1*, Blimp1*B Lymphocyte-Induced Maturation Protein 1*, RISC*RNA-Induced Silencing Complex*, Dicer*Double-stranded RNA-specific Endoribonuclease*, miRNA*MicroRNA*, pri-miRNA*Primary MicroRNA*, pre-miRNA*Precursor MicroRNA.

In this review, we summarized the current understandings of epigenetic regulation of crucial genes which play influential role in the differentiation, long-term persistence, and function of TRM. Several studies have demonstrated that three major kinds of epigenetics including DNA methylation, histone modifications and ncRNAs are all involved in the regulation of the biology of TRM (**Figure 1**). As a subset of T cells, CD4^+^and CD8^+^TRM retain many canonical features of their helper and cytotoxic lineages, sharing epigenetic traits with circulating T cells, yet displaying a distinct chromatin landscape. Their hallmark is tissue residency. TRM-specific loci such as *Cd69*, *Itgae*, and *Prf1*exhibit sustained hypomethylation and increased chromatin accessibility, facilitating tissue retention and cytotoxicity. In contrast, recirculation-associated genes like *S1pr1*and *Klf2*are epigenetically repressed. These stable epigenetic signatures underscore the non-recirculating identity of TRM (17).

### 6.1 Tissue-Specific Epigenetic Programming of TRM

TRM comprise diverse subsets that arise in distinct anatomical sites, where they are shaped by local environmental cues such as tissue-specific cytokines, metabolites, cellular interactions, and extracellular matrix components (3). Studies have shown that CD8^+^TRM exhibit both shared and tissue-specific transcriptional programs, with epigenetic and transcriptional profiles varying across non-lymphoid tissues such as the small intestine, kidney, liver, salivary glands, and adipose tissue. PageRank analysis further revealed tissue-specific transcriptional regulators, including Blimp1 in the small intestine, Ahr in the skin and liver, and Hic1 as a gut-restricted factor (17). However, whether these tissue-specific transcriptional regulators interact with epigenetic modifiers to potentially regulate the biology of TRM remains unexplored. In the following section, we summarize key transcription factors involved in TRM regulation across major tissues, aiming to provide insights for future investigations.

#### 6.1.1 Small intestinal TRM

Small intestinal TRM display the most distinct features following LCMV infection, characterized by uniquely accessible chromatin regions at the transcription start site of Ccr9, a key tissue residency marker (17). CCR9 facilitates the recruitment of activated CD8 αβ^+^effector T cells to the gut via its ligand CCL25, which is abundantly expressed in the small intestine (100). T-box transcription factors such as Eomes are more strongly downregulated in small intestinal TRM compared to those from other sites. Eomes serves as a negative regulator of TRM residency, while transforming growth factor β (TGF-β), a key conductor of the tissue residency program, particularly at epithelial barriers, modulates Eomes and promotes CD103 expression. Although Eomes inhibits TRM formation in certain tissues, yet exhibits unexpected, context-specific regulatory roles by supporting the maintenance of established TRM in the small intestine (20). In addition, Hobit and Blimp1 are essential transcriptional repressors for the formation of intestinal CD8^+^TRM. They are highly expressed in small intestinal intraepithelial TRM and cooperatively repress genes associated with tissue egress, including *Klf2*, *S1pr1*, *Ccr7*, and *Tcf7*. In T cell–specific Hobit and Blimp1 double-knockout mice, CD8^+^TRM failed to form in the gut despite normal effector expansion, thereby ameliorating disease severity in multiple experimental colitis models (42).

#### 6.1.2 Lung TRM

Lung CD8^+^TRM also exhibit distinct transcriptional regulation compared to TRM in other tissues. Unlike skin or intestinal TRM, lung TRM maintenance is independent of Hobit but critically dependent on Blimp1. Blimp1 promotes TRM fate by repressing TCF-1 expression and inhibiting TCM differentiation, thereby guiding lineage commitment toward the TRM program following influenza infection (101, 102). Moreover, Lung CD8^+^TRM do not require Hobit for their development and lack persistent granzyme B protein expression, unlike TRM in liver or intestine (103). This correlates with their reduced cytotoxicity. Since Hobit is essential for granzyme B maintenance in other tissues, its absence in lung TRM may underlie their limited effector function, highlighting a tissue-specific transcriptional regulation of TRM by Hobit and possibly Blimp1. Additionally, Extensive transcription factor and gene set enrichment analysis of lung TRM showed that, under inflammatory conditions, several drivers of the T cell effector function were overexpressed in these cells, such as Runx3, IRF4 and NF-kB (104).

#### 6.1.3 Liver TRM

Like their counterparts, liver TRM are also characterized by upregulation of Blimp1 and Hobit. Notably, Hobit expression in liver TRM has been shown to be regulated by the gene repressor Capicua in cooperation with the ETS variant transcription factor 5 (ETV5), highlighting the critical role of ETV5 in the transcriptional control of these cells (105). In the liver, TRM exhibit tissue-specific adaptations, particularly in response to the hypoxic microenvironment created by the organ’s venous blood supply and slow sinusoidal flow. These conditions induce the expression of hypoxia-inducible transcription factors HIF-1 and HIF-2, which are crucial for regulating T cell development, metabolism, and function under low oxygen levels. A distinct intrahepatic TRM subset characterized by CD69^+^CD103^-^HIF-2^+^expression has been identified, predominantly residing in hypoxic regions of the liver. Notably, this HIF-2^+^TRM population is absent in other tissues such as the lung, skin, or colon, highlighting the unique role of HIF-2 in mediating the liver-specific adaptation of TRM cells to hypoxic conditions (106).

#### 6.1.4 Skin TRM

In skin TRM, the transcription factors Runx2 and Runx3 play key roles in promoting the differentiation of cytotoxic CD8^+^CD103^+^CD49a^+^TRM from circulating memory T cell precursors. These Runx family members are highly expressed in epidermal TRM and drive a cytotoxic transcriptional program upon IL-15 and TGF-β stimulation. Runx2, in particular, is associated with enhanced TRM cell cytotoxicity and correlates with improved survival in melanoma patients, indicating its relevance for effective local immune surveillance (21).

### 6.2 Transcription Factors Linking Epigenetic Regulation in TRM

Although the transcription factors mentioned above play important roles in regulating tissue-specific TRM biology, there are limited reports exploring their interactions with epigenetic modifiers. However, several studies have reported such interactions between these transcription factors and epigenetic enzymes in T cells. Blimp1, one of the most extensively studied transcription factors involved in TRM regulation, has been shown to maintain Treg cell identity and function under inflammatory conditions by repressing Dnmt3a-mediated DNA methylation at the Foxp3 conserved non-coding sequence 2 (CNS2) locus, thereby preserving chromatin accessibility and sustaining Foxp3 expression (107). Whether Blimp1 similarly influences TRM biology through interactions with DNMTs remains to be determined. In CD8^+^T cells, Blimp1 has also been identified as an epigenetic regulator that directly represses *Il2ra*and *Cd27*by recruiting the histone-modifying enzymes G9a and HDAC2 during the peak of antiviral responses. This recruitment leads to increased H3K9 trimethylation and decreased histone H3 acetylation at these loci, thereby limiting cytokine responsiveness and promoting the differentiation of short-lived effector cells over memory precursors (108). Moreover, Blimp1 can epigenetically repress IL-21 expression in T cells by reducing chromatin accessibility and displacing the transcriptional activator c-Maf from the *Il21*promoter, thus establishing a negative feedback loop essential for immune homeostasis (109). These findings not only underscore the capacity of Blimp1 to function as a transcriptional repressor but also highlight its broader role as an epigenetic modulator that orchestrates T cell fate decisions via chromatin remodeling. Given the critical role of Blimp1 in enforcing TRM identity—particularly in the lung and gut—it is plausible that similar epigenetic mechanisms are employed to regulate TRM differentiation and maintenance in a tissue-specific manner. For instance, Blimp1-mediated recruitment of HDAC2 and G9a may contribute to the silencing of genes associated with tissue egress or central memory differentiation (e.g., *Klf2*, *S1pr1*, *Ccr7*, *Tcf7*), thereby promoting TRM residency. Likewise, its potential interaction with DNMTs could help establish and stabilize the TRM epigenetic landscape under inflammatory or homeostatic conditions. Although direct evidence of such epigenetic interactions in TRM is still lacking, the parallels drawn from conventional T cells suggest a promising avenue for future research. These examples highlight transcription factor–epigenetic crosstalk in T cells. We also summarize the transcription factors which interact with epigenetic modifiers in T cells (**Table 1**).

**Table 1:** Transcription factor–epigenetic regulator interactions in T lymphocytes.

Transcription factors	Epigenetic interactions in T cells	Reference
Bcl11b	Bcl11b maintains multipotency in intestinal CD8^+^TRM by enhancing H3K27ac, H3K4me3, and chromatin accessibility at promoters of Tcf7 and Id3 while repressing effector gene expression	(46)
Osr2	Osr2 recruits HDAC3 to suppress cytotoxic genes and drive CD8^+^T cell exhaustion	(94)
Ikzf1	Foxp3–Ikzf1–Ikzf3 complex repress pro-inflammatory genes in Treg by competing with p300 at target loci.	(95)
Runx3	Runx3 binds chromatin in naive CD8^+^T cells and is required during TCR stimulation to open memory-CTL-specific cis-regulatory regions, enabling accessibility for IRF, bZIP, and Prdm1 motifs.	(96)
BATF	BATF promotes CD8^+^T cell effector differentiation by repressing Sirt1, a NAD^+^-dependent histone deacetylase, thereby enhancing histone acetylation at the T-bet locus.	(97)
RFX1	RFX1 represses CD70 and CD11a expression in CD4^+^T cells by recruiting DNMT1 and HDAC1; its loss in SLE leads to epigenetic upregulation of these activation markers	(98)
Foxp3	Foxp3 represses gene expression in Treg cells via Tip60, HDAC7, and Eos; loss of these interactions reduces acetylation and impairs function.	(99)

### 6.3 Microenvironment-Driven Epigenetic Diversity of TRM

**Figure 2:**
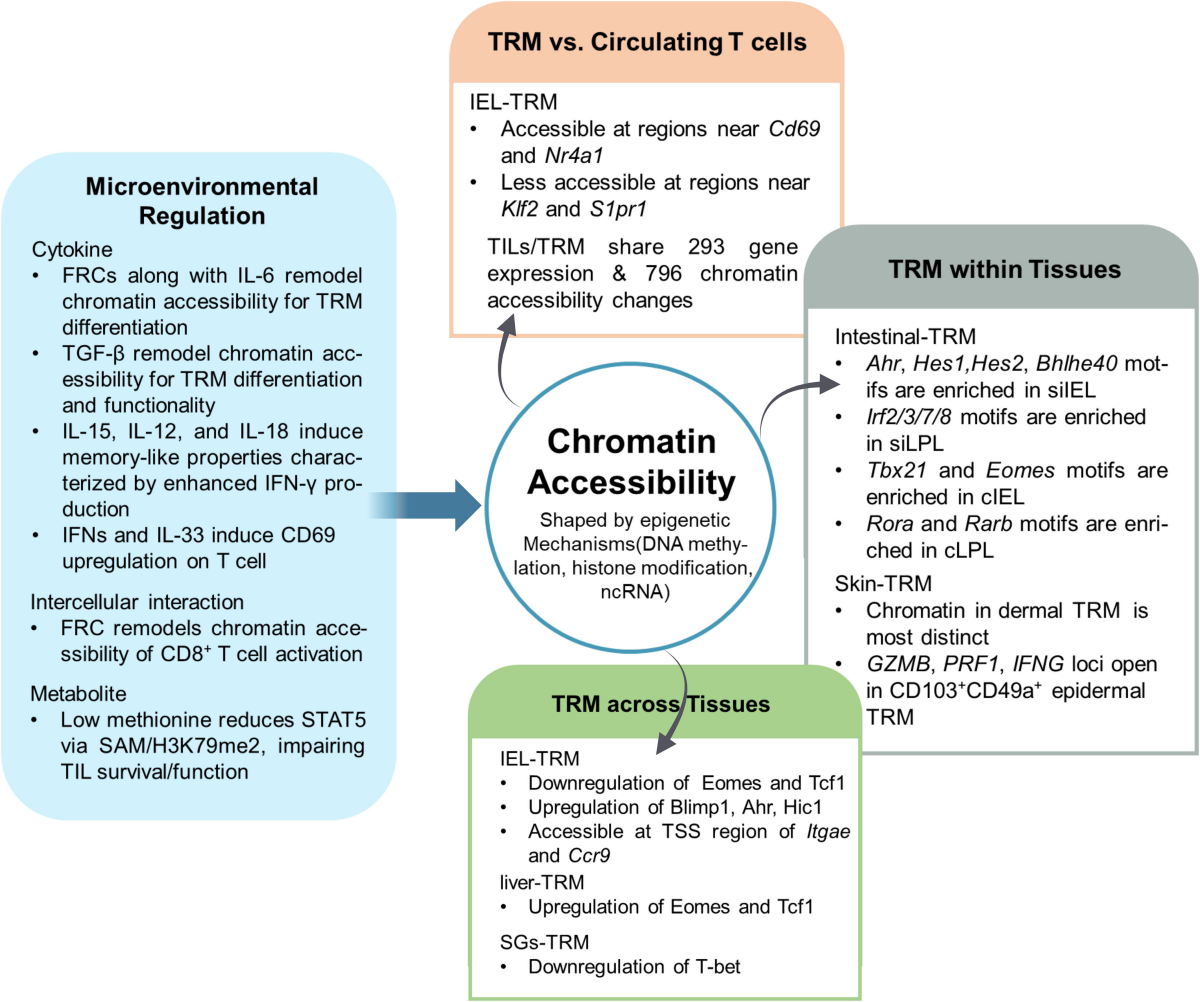
Tissue microenvironment-driven epigenetic heterogeneity of tissue resident memory T cells. The tissue microenvironment profoundly influences the chromatin accessibility landscape of TRM, resulting in both inter- and intra-tissue heterogeneity. *FRCs*Fibroblastic reticular cells, *IL*Interleukin, *TGF-β*Transforming growth factor β, *STAT5*Signal transducer and activator of transcription 5, *SAM*S-adenosylmethionine, *TIL*Tumor-infiltrating lymphocyte, *Nr4a1*Nuclear receptor subfamily 4 group A member 1, Klf2 Kruppel-like factor 2, S1pr1 Sphingosine-1-phosphate receptor 1, *Ahr*Aryl hydrocarbon receptor, *Hes1/2*Hairy and enhancer of split-1/2, *Bhlhe40*Basic helix-loop-helix family member e40, *Tbx21*T-box transcription factor 21, *Eomes*Eomesodermin, *Rora*RAR-related orphan receptor alpha, *Rarb*Retinoic acid receptor beta, *Tcf1*T cell factor 1, *Blimp1*B lymphocyte-induced maturation protein 1, *Itgae*Integrin subunit alpha E, *Ccr9*C-C motif chemokine receptor 9, *siIEL*Small intestine intraepithelial lymphocyte, *siLPL*Small intestine lamina propria lymphocyte, *cIEL*Colon intraepithelial lymphocyte, *cLPL*Colon lamina propria lymphocyte, *TSS*Transcription start site, *T-bet*T-box transcription factor.

Due to the heterogeneity of TRM, it is important to understand how microenvironmental cues orchestrate their epigenetic and transcriptional profiles. Epigenetic regulation of TRM is shaped by multiple upstream signals from the tissue microenvironment (**Figure 2**). In addition to the FRC-mediated chromatin remodeling during early CD8^+^T cell activation mentioned above, it has been found that TRM can be regulated by cytokines, transcription factors and metabolites (12). For example, TGF-β and interleukins have been shown to promote the differentiation and functionality of TRM (110, 111). And they were all reported regulating the downstream genes epigenetically. Studies have shown that TGF-β, together with bone morphogenetic protein, binds to Type II receptors, initiating receptor complex formation and sequential activation of Type I receptors. The GS domain of the Type I receptor then phosphorylates receptor-regulated SMADs (R-SMADs), which subsequently associate with Co-SMADs to form an active SMAD complex. Upon nuclear translocation, this complex recruit various epigenetic regulators—such as histone-modifying enzymes (HATs, HDACs, KDMs, Suv39h, EZH2), DNA methylation modifiers (DNMTs, TDG), chromatin remodelers (SWI/SNF complex), and lncRNAs (DIGIT)—to modulate the transcription of context-dependent TGF-β target genes. This recruitment is not uniform across all loci. SMAD complexes can cooperate with SWI/SNF chromatin remodeling complexes to facilitate nucleosome displacement at target promoters or interact with HDAC-containing repressor complexes to restrict gene expression. Notably, activin-mediated signaling has been shown to reduce EZH2 protein levels through SMAD2, resulting in decreased H3K27me3 and facilitating transcriptional activation during differentiation processes (112). Together, these findings support a model in which cytokine receptor engagement initiates signaling cascades that converge on the recruitment or modulation of chromatin remodeling enzymes and histone/DNA modifiers to specific loci, establishing stable epigenetic landscapes that underpin cell identity and function (113). Other cytokines like type I interferons (IFNs) and IL-33 could induce CD69 upregulation on T cells (114, 115). IFNs lead to the transcription of IFN-stimulated genes in various immune cells such as T cells and macrophages by activating the JAK/STAT pathway (116). This facilitation of transcription was mainly due to the alteration of epigenetic landscape by the negative histone mark histone H3 lysine 9 dimethylation, which attenuates gene expression (117). IL-33 was found to ameliorate Aβ pathology by reprogramming microglial epigenetic profiles in Alzheimer’s Disease (118). In addition, KLF2, a transcription factor which is downregulated in TRM to promote the formation of CD69 was found it underlies the regeneration and persistence of a subpopulation of miRNA -125^High^breast tumor cells via an epigenetic way (19, 119, 120). Sustained BATF expression promotes the formation of CD69^+^CD103^+^tumor-infiltrating lymphocytes, which depends on the downregulation of KLF2 (19). Furthermore, the changes of metabolites also influence the epigenetics of cells (12). For example, the absence of methionine impairs the survival and function of tumor-infiltrating lymphocytes by decreasing s-adenosylmethionine levels, which subsequently reduces the expression of signal transducer and activator of transcription 5 through its impact on dimethylation at lysine 79 of histone H3 (121). Cellular regulation is a multifaceted and dynamic process involving numerous molecular pathways. Collectively, these examples indicate that various TRM regulatory factors—such as cell–cell interaction–mediated signals, cytokines, transcription factors and metabolites—can shape the epigenetic landscape of diverse cell types, leading to the emergence of TRM-like features. However, the identification of common epigenetic regulators that govern TRM biology remains largely unexplored and warrants further investigation as potential therapeutic targets.

### 6.4 Epigenetic Targeting of TRM for Therapy and Beyond

Over past decades, interventions targeting TRM in the context of disease have increasingly been brought to the spotlight, because of the unique characteristics of TRM in antitumor surveillance and pathogen resistance. However, none of methods were verified accurately regulating the TRM. In terms of tumor immunotherapy, application of anti-programmed death ligand 1 therapy can boost the infiltration of TRM in tumor (122). Radiotherapy and vaccination have also been shown to be beneficial for TRM survival, thereby exerting their antitumor properties (123). Although these therapies are effective in antitumor treatment, non-response and adverse effects in certain patients limit their broader clinical utility. Additionally, current approaches face critical challenges including off-target effects, lack of tissue specificity in drug delivery, and inter-individual heterogeneity in TRM response profiles. Given that TRM reside in diverse tissue microenvironments with distinct molecular and metabolic contexts, developing therapies that can selectively modulate TRM without affecting other resident or circulating immune populations remains a major obstacle. Furthermore, differences in TRM differentiation states, epigenetic landscapes, and responsiveness to cytokines across tissues contribute to heterogeneous therapeutic outcomes. As far as viral immunity, TRM can trigger the innate and adaptive responses through cytokine secretion thus promote activation of dendritic cells and recruitment of CD8^+^circulating memory T cells (124, 125). Meanwhile, TRM have an independent effect on cell function through the release of granzyme B and perforin when rechallenged by viruses (126). Therefore, vaccines targeting TRM have the potential to resist viral invasion. On the contrary, the excessive accumulation of malfunctional TRM leads to occurrence of chronic inflammatory diseases. The formation, residence, and function of TRM in the aforementioned diseases have changed both before and after treatment, indicating that therapies targeting TRM may be a valuable and promising option.

Epigenetic therapy has long been applied in immune-oncology (127). This is not only due to the epigenetic alterations occurring within the tumor itself but also because the infiltrating immune cells exhibit epigenetic abnormalities (128, 129). Clinical evidence shows that epigenetic therapies have achieved greater success in hematologic malignancies such as acute myeloid leukemia and myelodysplastic syndromes, where DNA methylation inhibitors like azacitidine and decitabine have demonstrated durable responses and are widely approved (130, 131). In contrast, their efficacy in solid tumors remains limited, potentially due to tumor heterogeneity, poor drug penetration, and the lack of well-defined epigenetic drivers. Nevertheless, several studies have indicated that combining epigenetic therapies with cytotoxic or targeted treatments can enhance chemosensitivity and improve outcomes in certain solid tumors, including non-small cell lung cancer, ovarian cancer, and breast cancer (132, 133). Except for DNA methylation inhibitors, histone deacetylase inhibitors (HDACi) are now the mainstay of therapies forcutaneous T cell lymphoma (CTCL) (134–137). Although the precise mechanisms of action for epigenetic drugs remain unclear due to the complexity of epigenetic alterations within the tumor microenvironment, the use of HDACi in patients with CTCL offers promising potential for similar therapeutic approaches in diseases characterized by TRM (138). This is particularly relevant because CTCL shares an epigenetic profile with TRM, as the disease arises predominantly in the skin through the clonal expansion of transformed TRM (139). Epigenetic modifications rarely function in isolation; rather, they typically interact within a complex network that governs the broader epigenetic landscape (140). As a result, medications targeting these systems are often considered “broad reprogrammers” that induce widespread changes across the epigenome. While these agents have demonstrated some therapeutic efficacy, they are not ideal due to their generalized effects on the epigenetic network (141). In contrast to broad reprogrammers, targeted therapies addressing specific genetic alterations have gradually gained more attention. They have potential to control the fate commitment and function of TRM, thereby providing therapeutic opportunities not only for the treatment of malignancies but also for inflammatory disorders. For instance, EZH2 inhibitors have been shown to repress TRM differentiation, thereby alleviating inflammatory diseases (43). Despite the growing interest in TRM biology, targeted epigenetic interventions specifically designed to modulate TRM function remain largely unexplored. Nevertheless, based on the distinctive features of TRM summarized in this review, targeted strategies may be developed by leveraging their tissue-specific transcriptional programs. One possible approach is to selectively deliver epigenetic modulators to TRM using tissue-specific promoters, nanoparticle-based carriers, or ligand–receptor systems that recognize TRM-enriched surface markers such as CD69 or CD103. Alternatively, bifunctional molecules designed to simultaneously engage TRM-specific transcription factors (e.g., Runx3, Blimp1) and epigenetic enzymes (e.g., EZH2, DNMTs) may offer a promising strategy to achieve cell-type-restricted epigenetic modulation, thereby enhancing therapeutic efficacy while minimizing off-target effects. Taken together, research on epigenetic changes in TRM is still in its early stages, and further investigation is needed to fully elucidate their regulatory mechanisms. Integrating epigenetic regulatory medications with the modulation of TRM represents a promising emerging strategy for disease treatment.

## 7 Conclusion

Collectively, current evidence establishes that epigenetic regulation is integral to the formation, persistence, and functional specialization of tissue-resident memory T cells. Chromatin remodeling, DNA methylation, histone modifications, and non-coding RNAs converge with tissue-specific cues to define TRM identity and heterogeneity. Despite these advances, key gaps remain in understanding how dynamic epigenetic landscapes are orchestrated during TRM differentiation and recall responses. Addressing these questions will benefit from single-cell multi-omic approaches, lineage tracing, and CRISPR-based epigenome editing to resolve causal mechanisms with spatial and temporal precision. Therapeutically, selective modulation of TRM epigenetic programs represents a promising strategy to augment antitumor immunity, enhance vaccine-induced protection, and attenuate pathogenic TRM activity in autoimmunity and chronic inflammation.
